# Response to Immune Checkpoint Inhibitor Therapy in Patients with Unresectable Recurrent Malignant Pleural Mesothelioma Shown by FDG-PET and CT

**DOI:** 10.3390/cancers13051098

**Published:** 2021-03-04

**Authors:** Kazuhiro Kitajima, Mitsunari Maruyama, Hiroyuki Yokoyama, Toshiyuki Minami, Takashi Yokoi, Akifumi Nakamura, Masaki Hashimoto, Nobuyuki Kondo, Kozo Kuribayashi, Takashi Kijima, Seiki Hasegawa, Koichiro Yamakado

**Affiliations:** 1 Department of Radiology, Hyogo College of Medicine, 1-1 Mukogawa-cho, Nishinomiya 663-8501, Japan; mit-maruyama@hyo-med.ac.jp (M.M.); yokoyama.h@meiwa-hospital.com (H.Y.); yamakado@hyo-med.ac.jp (K.Y.); 2 Department of Internal Medicine, Division of Respiratory Medicine, Hyogo College of Medicine, 1-1 Mukogawa-cho, Nishinomiya 663-8501, Japan; to-minami@hyo-med.ac.jp (T.M.); ta-yokoi@hyo-med.ac.jp (T.Y.); kuririn@hyo-med.ac.jp (K.K.); tkijima@hyo-med.ac.jp (T.K.); 3 Department of Thoratic Surgery, Hyogo College of Medicine, 1-1 Mukogawa-cho, Nishinomiya 663-8501, Japan; ak-nakamura@hyo-med.ac.jp (A.N.); masaki-h@hyo-med.ac.jp (M.H.); kondon@hyo-med.ac.jp (N.K.); hasegawa@hyo-med.ac.jp (S.H.)

**Keywords:** mesothelioma, immunotherapy, therapy response, survival, FDG, PET-CT

## Abstract

**Simple Summary:**

This is the first known study to compare three FDG-PET/CT criteria (EORTC, PERCIST, imPERCIST) with CT criteria (combined modified RECIST and RECIST 1.1) used to evaluate tumor response to ICI therapy in patients with recurrent MPM as well as prediction of prognosis. All of the FDG-PET/CT and CT criteria analyzed were found to be accurate for both evaluation of tumor response and prediction of progression free survival in the present cohort. In comparison with CT, all three FDG-PET/CT criteria judged a greater percentage of patients (16.7%) as CR, while two (EORTC, PERCIST) judged a greater percentage (10–13.3%) as PD.

**Abstract:**

Background: To compare three FDG-PET criteria (EORTC, PERCIST, imPERCIST) with CT criteria (combined modified RECIST and RECIST 1.1) for response evaluation and prognosis prediction in patients with recurrent MPM treated with ICI monotherapy. Methods: Thirty MPM patients underwent FDG-PET/CT and contrast-enhanced CT at the baseline and during nivolumab therapy (median 10 cycles). Therapeutic response was evaluated according to EORTC, PERCIST, imPERCIST, and CT criteria. PFS and OS were examined using log-rank and Cox methods. Results: CMR/PMR/SMD/PMD numbered 5/3/4/18 for EORTC, 5/1/7/17 for PERCIST, and 5/3/9/13 for imPERCIST. With CT, CR/PR/SD/PD numbered 0/6/10/14. There was high concordance between EORTC and PERCIST (κ = 0.911), and PERCIST and imPERCIST (κ = 0.826), while that between EORTC and imPERCIST (κ = 0.746) was substantial, and between CT and the three PET criteria moderate (κ = 0.516–0.544). After median 14.9 months, 26 patients showed progression and nine died. According to both PET and CT findings, patients with no progression (CMR/PMR/SMD or CR/PR/SD) showed significantly longer PFS and somewhat longer OS than PMD and PD patients (EORTC *p* = 0.0004 and *p* = 0.055, respectively; PERCIST *p* = 0.0003 and *p* = 0.052; imPERCIST *p* < 0.0001 and *p* = 0.089; CT criteria *p* = 0.0015 and *p* = 0.056). Conclusions: Both FDG-PET and CT criteria are accurate for response evaluation of ICI therapy and prediction of MPM prognosis. In comparison with CT, all three FDG-PET/CT criteria judged a greater percentage of patients (16.7%) as CMR, while two (EORTC, PERCIST) judged a greater percentage (10–13.3%) as PMD. For predicting PFS, the three FDG-PET criteria were superior to the CT criteria, and imPERCIST demonstrated the highest rate of accurate prediction.

## 1. Introduction

Individuals affected by malignant pleural mesothelioma (MPM), a rare type of aggressive malignancy, have a poor prognosis. Platinum-based chemotherapy has been commonly used as the standard first-line treatment in unresectable MPM cases, though few other treatment options are available for those not showing response. However, a paradigm shift has occurred in recent years because of development of immune checkpoint inhibitors (ICIs), and several groups have reported survival benefits for patients with recurrent MPM [1,2,3,4,5]. Those include a single-arm phase II study conducted in Japan (MERIT study) that examined nivolumab (anti-PD-1 monoclonal antibody) monotherapy for efficacy and safety in 34 MPM patients with a history of chemotherapy, with their findings leading to approval of nivolumab for unresectable recurrent MPM treatment in Japan [3].

A crucial factor for effective cancer treatment management is adequate assessment of systemic treatment response, with efficient monitoring of responsiveness to systemic therapy by the tumor vital for moderating the high risk of mortality and also cytotoxic effects associated with systemic therapeutic regimens. Classic methods have been developed for examining patients undergoing cytotoxic chemotherapy and given molecular targeted agents are used for evaluation of treatment response, such as the Response Evaluation Criteria in Solid Tumors version 1.1 (RECIST 1.1) [6] for computed tomography (CT), and the European Organization for Research and Treatment of Cancer (EORTC) criteria [7] and Positron Emission Tomography Response Criteria in Solid Tumors (PERCIST) [8] for [^18^F]fluorodeoxyglucose positron emission tomography/computed tomography (FDG-PET/CT), as those treatments can directly result in reduced tumor cell viability. However, immunotherapy differs from classical cytotoxic drugs in regard to the action mechanism, as that mechanism of the former is based on stimulation of host immune response against cancer cells, possibly resulting in inflammation development at the tumor site, leading to a subsequent antitumor response [9].

ICI therapeutic efficacy is difficult to assess and the role of FDG-PET has not yet been established. An increase in FDG uptake or appearance of new lesions following therapy may represent infiltration of cancer foci by host immune cells (pseudo-progression) rather than true tumor progression, thus making evaluation of treatment response using FDG-PET/CT results challenging. As a result, another group recently proposed immunotherapy-modified PERCIST (imPERCIST) findings for this evaluation, in which new lesions are not considered to define progressive metabolic disease (PMD) during the early period of assessment (2–4 cycles) of ICI response in metastatic melanoma patients [10].

No other known studies have examined or compared use of FDG-PET/CT and CT for determining MPM patient response to ICI therapy. The present retrospective investigation compared three functional FDG-PET criteria (EORTC, PERCIST, imPERCIST) with morphological CT criteria (combined modified RECIST [11] and RECIST 1.1 [6]) to evaluate response to treatment and predict prognosis in patients with recurrent MPM undergoing nivolumab monotherapy treatment.

## 2. Materials and Methods

### 2.1. Patients

Approval from a local review board was received for this retrospective study, and the requirement for patient-informed consent was waived. A search of our database was used to obtain the records of patients with unresectable recurrent MPM and treated with nivolumab monotherapy between June 2018 and December 2019. For the present analysis, a total of 30 (mean 68.1 ± 7.2 years old, range 46–77 years) who underwent FDG-PET/CT and contrast-enhanced CT examinations at our institution at the baseline and during nivolumab monotherapy (after 4–6 cycles in 3, 7–9 in 9, 10–12 in 9, 13–15 in 4, 16–18 in 3, 19–21 in 2; median 10 cycles) for treatment response evaluation were included. Baseline FDG-PET/CT and baseline contrast-enhanced CT examinations were conducted at a median 1.0 months (1.0–2.2 months) and 1.4 months (0.7–2.3 months), respectively, before initiation of nivolumab therapy. The interval of FDG-PET/CT and contrast-enhanced CT was less than two weeks at the baseline and during nivolumab therapy in every patient. Table 1 shows patient and tumor characteristics. CT, FDG-PET/CT, and brain magnetic resonance imaging (MRI) results were used for diagnosis of disease recurrence, metastasis, and progression during the follow-up period. When disease progression or recurrence was suspected on the physical findings, CT or FDG-PET/CT was undertaken for the evaluating the whole-body state, and the brain MRI was carried out for the screening of the brain. In some patients without suspected progression or recurrence, those imaging examinations were undertaken every 6–12 months for surveillance.

Intravenous nivolumab was given at 3 mg/kg every two weeks until apparent disease progression or unacceptable toxicity was observed, or the patient or attending physician decided to discontinue treatment. Of the 30 enrolled patients, treatment-related adverse events were noted in nine (30.0%) (rash in two, hypothyroidism in two, interstitial lung disease in one, increased lipase level in one, diarrhea in one, hypoadrenocorticism in one, fatigue in one). After discontinuing nivolumab treatment, alternative treatment (cisplatin/carboplatin and pemetrexed, pemetrexed, or irinotecan and gemcitabine) was tried.

### 2.2. FDG-PET/CT

Four different PET/CT scanners installed at our institution (Gemini GXL16, Gemini TF64, Ingenuity TF: Philips Medical Systems, Eindhoven, The Netherlands; Discovery IQ: GE Healthcare, Waukesha, WI, USA) were used for performing the FDG-PET/CT examinations. Each patient was instructed to fast for five hours before the examination, and blood glucose was measured immediately prior to FDG injection (4.0 MBq/kg body weight for GXL16, 3.0 MBq/kg for TF64, 3.7 MBq/kg body weight for Ingenuity TF and Discovery IQ), with all in the present cohort showing a level lower than 160 mg/dL. Approximately 60 min after the injection, static emission images were obtained. For attenuation correction and anatomic localization, helical CT scan images from the top of the head to mid-thigh were obtained with the following parameters: tube voltage 120 kV (all four scanners), effective tube current auto-mA up to 120 mA (GXL16), 100 mA (TF64), 155 mA (Ingenuity TF), or 15–390 mA (Smart mA: noise index 25) (Discovery IQ), gantry rotation speed 0.5 s, detector configuration 16 × 1.5 mm (GXL16), 64 × 0.625 mm (TF64 and Ingenuity TF), or 16 × 1.25 mm (Discovery IQ), slice thickness 2 mm, and a transverse field of view 600 mm (GXL16, TF64, Ingenuity TF) or 700 mm (Discovery IQ). Immediately after completion of the CT examination, PET imaging was performed from the head to mid-thigh for 90 s (GXL16, TF64, Ingenuity TF) or 180 s (Discovery IQ) per bed position in three-dimensional mode. The patient was allowed to breathe normally during PET scanning. For the GXL16, attenuation-corrected PET images were reconstructed with a line-of-response row-action maximum likelihood algorithm, while for the TF64 and Ingenuity an ordered-subset expectation maximization (OSEM) iterative reconstruction algorithm (33 subsets, three iterations) was used, and Q.Clear (block sequential regularized expectation maximization (BSREM)) (β = 400) was utilized for the Discovery IQ.

### 2.3. Contrast-Enhanced CT

To obtain pre-contrast and contrast-enhanced CT images of the neck, chest, abdomen, and pelvis, a 128-detector row CT (SOMATOM Definition AS: Siemens Healthcare, Erlangen, Germany) was used at 120 kV, with an effective mA of 220 (CAREDose4D), beam pitch of 0.6, collimation of 1.2 × 32 mm, and B31 + medium smooth + image reconstruction. Details regarding the contrast-enhanced CT procedures have been previously presented. Briefly, blood creatinine level determined prior to the examination was ≤1.5 mg/dL in all of the patients. Iodinated contrast material (Iopamiron Inj, Syringe, Bayer Schering Pharma, Berlin, Germany) containing 300 mg of iodine per ml at a dose of 600 mg of iodine per kg of body weight was intravenously administered using a power injector, with scanning started at 120 s after the injection.

### 2.4. Image Analysis

A board-certified nuclear medicine expert with 12 years of oncologic FDG-PET/CT experience and without knowledge of the other imaging results, or clinical or histopathologic data for the present patients, retrospectively reviewed the FDG-PET/CT images. To assist the attending clinician with treatment response monitoring, the GI-PET software package (AZE Co., Ltd., Tokyo, Japan), which can harmonize standardized uptake values (SUVs) obtained with different PET/CT systems using phantom data [12], was employed. Maximum SUV (SUVmax) was defined as the maximum concentration in the target lesion (injected dose/body weight). For calculating SUVpeak, a 1.2-cm diameter volume region of interest (ROI) placed on the hottest site of the tumor was used, then normalized to SUV corrected for lean body mass (SULpeak) (SUVpeak × [lean body mass]/[total body mass]).

A board-certified radiologist with 12 years of experience with CT retrospectively evaluated the contrast-enhanced CT images and made determinations, in the absence of knowledge of the other imaging results or clinical data for the present patients. Coronal, axial, and sagittal section images were viewed and analyzed, with appropriate winding applied.

### 2.5. EORTC

Using the EORTC guidelines [7], complete resolution of FDG uptake within the tumor volume indistinguishable from surrounding normal tissue was determined as complete metabolic response (CMR), while PMD was the classification for appearance of new FDG uptake in another region in the second FDG-PET/CT scan. The EORTC recommends defining regions of high FDG uptake that represent a viable tumor by use of pre-treatment scan findings and also utilization of the same ROI volumes in subsequent scanning examinations positioned as close to the original tumor as possible, as well as determination of maximal tumor ROI count per pixel per second calibrated as MBq/L [7]. The number of lesions to be measured is not recommended by the EORTC, thus up to five with the highest level of FDG uptake and up to two per organ, with same lesions measured in subsequent follow-up scan imaging results, were the parameters used in the present study [13]. The values for all five targets used for SUVmax measurement were summed for each scan, resulting in ΣSUVmax. Percentage changes from baseline to second summed SUVmax were calculated, with a reduction of ≥25% in summed SUVmax value defined as partial metabolic response (PMR). PMD was classified as an increase in tumor summed SUVmax value ≥25% within the ROI defined based on the baseline scan, while stable metabolic disease (SMD) was defined as an increase in the summed SUVmax value of <25% or a decrease <25%.

### 2.6. PERCIST

For therapeutic response determination according to PERCIST [8], SUL values were calculated using a 1.2-cm diameter volume ROI placed on the target lesion, and SUL values were calculated. Additionally, the SULpeak value of the tumor was determined and noted if it was 1.5 times or more greater than that of the liver SUL (mean ± 2 standard deviations) in a 3-cm diameter spherical ROI on the normal right lobe. When complete resolution of FDG uptake within the target lesion was lower than mean liver activity and indistinguishable from the background blood-pool level, CMR was the classification. For cases with metabolically active lesions noted in follow-up scan findings, the SULpeak of up to five lesions at the baseline and follow-up examinations was summed (maximum two per organ) [8]. The hottest lesions in each scan were selected; thus, the target lesions detected in follow-up imaging were not necessarily the same as those in the baseline images. When the SULpeak sum was decreased by ≥30%, tumor response for that case was classified as PMR. Conversely, an increase in SULpeak sum ≥30% or appearance of new hypermetabolic lesions or ≥75% increase in total lesion glycolysis (TLG) in follow-up FDG PET/CT scan imaging was defined as PMD. Any cases not defined as CMR, PMR, or PMD were classified as SMD.

### 2.7. imPERCIST

imPERCIST was performed in the same manner as used for PERCIST, though appearance of new lesions alone did not result in a classification of PMD [10], as that was defined only by increase in sum of SULpeaks of ≥30%. New lesions were included in the SULpeak sum when a higher uptake level than the existing target lesions was shown or when fewer than five target lesions were detected in baseline scan results.

### 2.8. Combined Modified RECIST and RECIST 1.1

Pleural tumor thickness perpendicular to the chest wall or mediastinum was measured at two different points at three different levels for evaluations with modified RECIST [11]. For assessing the morphological response of nonplural lesions, RECIST 1.1 was used [6]. The target lesion was defined as a well-defined soft tissue lesion with the longest axis for the lymph node ≥ 1 cm and the shortest axis ≥1.5 cm, and the greatest sum of the diameter of five target lesions, maximum two lesions per organ, and used for evaluation. Sclerotic or lytic/sclerotic (mixed type) bone metastasis was considered to be a non-measurable lesion. With both modified RECIST and RECIST 1.1, a decrease ≥30% in largest diameter sum was considered to be partial response (PR), while progressive disease (PD) was determined in cases with an increase ≥20%. Stable disease (SD) was considered to be any change between PR and PD of <−30% to <+20%; complete response (CR) was determined in cases with disappearance of nonplural target lesions and lymph nodes in the shortest axis <1 cm, and PD when there was appearance of a new lesion. In a comparison of mRECIST and RECIST 1.1 results, the worst objective response was chosen as the final classification shown by CT.

### 2.9. Statistical Analysis

Cohen’s κ coefficient was used to examine concordance between criteria methods was assessed using [14], with a slight (κ < 0.21), fair (κ = 0.21–0.40), moderate (κ = 0.41–0.60), substantial (κ = 0.61–0.80), or nearly perfect (κ > 0.80) level of agreement noted. Progression-free survival (PFS) was defined based on time elapsed from start of nivolumab therapy to date of disease progression shown in radiological and/or clinical examination results, or death from any cause. Any patient with no evidence of progressive disease was censored at the date of the last follow-up examination. Time from start of nivolumab therapy until death from any cause was used to determine overall survival (OS). Patients living at the final follow-up examination were censored, and classified as alive with disease or no evidence of progression. The Kaplan–Meier method was used to generate actuarial survival curves, with a log-rank test employed to examine differences between groups. Statistical analyses were performed with the SAS software package, version 9.3 (SAS Institute Inc., Cary, NC, USA), with *p* values < 0.05 considered to be significant.

## 3. Results

### 3.1. Treatment Response Assessment

**Figure 1:**
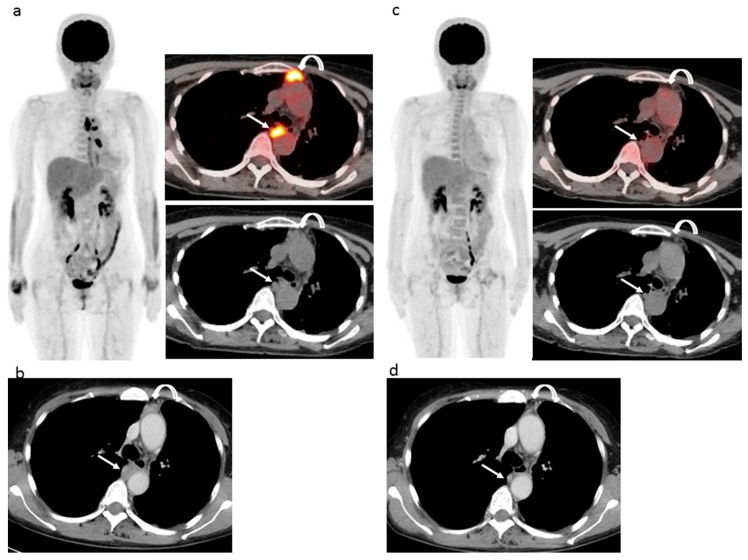
61 year-old woman with left epithelioid malignant pleural mesothelioma who previously received neoadjuvant chemotherapy (pemetrexed + cisplatin), pleurectomy, and decortication surgery (pT3N1M0), then six cycles of chemotherapy (pemetrexed + cisplatin) after the operation, followed by 10 cycles of second-line therapy (irinotecan + gemcitabine) and then nivolumab as third-line chemotherapy. (**a**) Pre-nivolumab treatment FDG-PET/CT shows several areas of strong FDG uptake related to a pleural lesion (curved arrow) and mediastinal lymph nodal lesion (arrow). (**b**) Pre-nivolumab treatment contrast-enhanced CT shows mass-forming thickness of pleura lesion (curved arrow) and mediastinal lymph nodal lesion (arrow). (**c**) During-treatment FDG-PET/CT after 13 cycles of nivolumab shows FDG uptake disappearance in both pleural (curved arrow) and nodal (arrow) lesions. (**d**) During-treatment contrast-enhanced CT after 13 cycles of nivolumab shows remarkable improvements of both pleural (curved arrow) and nodal (arrow) lesions. EORTC, PERCIST, and imPERCIST indicated CMR. Interpretation of combined modified RECIST and RECIST 1.1 indicated a classification of PR, with the sum pleural lesion size decreasing by 45.5% and the sum mediastinal node size decreasing by 78.3%. The patient continued with 29 more cycles of nivolumab and was alive without progression at 15.1 months after nivolumab initiation.

**Figure 2:**
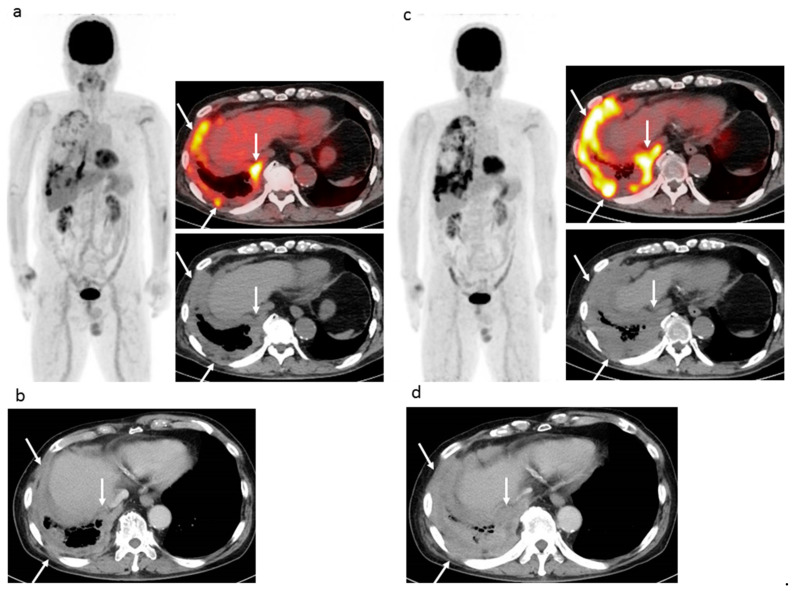
74 year-old man with right epithelioid malignant pleural mesothelioma (cT2N0M0), who previously received six cycles of first-line chemotherapy (pemetrexed + cisplatin) and then 12 cycles of nivolumab as second-line chemotherapy. (**a**) Pre-nivolumab treatment FDG-PET/CT shows multiple areas of strong FDG uptake in areas of right pleural lesions (arrows). (**b**) Pre-nivolumab treatment contrast-enhanced CT shows mass-forming thickness of right pleura (arrows). (**c**) Post-treatment FDG-PET/CT after 12 cycles of nivolumab shows remarkable progression of multiple pleural lesions (arrows) and appearance of new pleural lesions. (**d**) Post-treatment contrast-enhanced CT after 12 cycles of nivolumab shows remarkable progression of pleural lesions (arrows). EORTC, PERCIST, imPERCIST, and CT criteria (modified RECIST and RECIST 1.1) indicated PMD or PD due to remarkable progression and appearance of new lesions. In FDG-PET/CT results, the SULpeak sum of the five highest level pleural lesions was increased by 98.6%. In CT findings, the sum size of six pleural lesions perpendicular to the chest wall was increased by 40.3%. According to the second (**c**) FDG-PET/CT and (**d**) contrast-enhanced CT result, the patient started another chemotherapy series (irinotecan + gemcitabine), though was alive at 13.9 months after initiation of nivolumab.

Using EORTC criteria with FDG-PET/CT findings resulted in CMR being noted in five patients (16.7%), PMR in three (10.0%), SMD in four (13.3%), and PMD in 18 (60.0%), while use of PERCIST with FDG-PET/CT findings showed CMR in five (16.7%), PMR in one (3.3%), SMD in seven (23.3%), and PMD in 17 (56.7%) patients, respectively, and use of imPERCIST with FDG-PET/CT findings showed CMR in five (16.7%), PMR in three (10.0%), SMD in nine (30.0%), and PMD in 13 (43.3%) patients, respectively. When the combination of modified RECIST and RECIST 1.1 with CT was used, no patients (0%) had CR, six (20.0%) had PR, 10 (33.3%) had SD, and 14 (46.7%) had PD. Figure 1 and Figure 2 present data of two representative cases.

Prior to nivolumab treatment, FDG-PET/CT examinations showed only pleural lesions in 25 patients, while two had pleural and nodal lesions, one had only nodal lesions, one had pleural and lung lesions, and one had pleural, nodal, and peritoneal lesions. Tiny nodal or peritoneal lesions were not detected with contrast-enhanced CT in two patients before starting nivolumab treatment, though those are not included as target lesions in the RECIST criteria due to their small size. The second FDG-PET/CT examination detected new lesions in eight patients (lung metastasis in two; pleural lesions in one; lymph node metastasis in one; bone metastasis in one; small intestine metastasis in one; lymph node and peritoneal dissemination in one; lymph node, peritoneal, bone, and muscle metastasis in one). Of those eight cases with new lesions revealed in the second FDG-PET/CT examination, the CT reader was unable to detect new lesions in three (bone metastasis in one; small intestine metastasis in one; lymph node, peritoneal, bone, and muscle metastasis in one).

### 3.2. Treatment Response Assessment Comparisons among Criteria Methods

Twenty-seven (90%) of the cases demonstrated concordance between the EORTC criteria and PERCIST response classifications, while discordance was noted in three (10.0%), with nearly perfect agreement (κ = 0.911) for response classification between them (Table 2). As for EORTC and imPERCIST, concordance between them was seen in 23 (76.7%) cases and discordance was noted in seven (23.3%), with substantial agreement (κ = 0.746) for response classification found between them (Table 3). Furthermore, in 26 (86.7%) cases, concordance between PERCIST and imPERCIST was seen, and discordance was noted in four (13.3%), with nearly perfect agreement (κ = 0.826) for response classification found between them (Table 3). Four PMD patients defined by PERCIST were classified as SMD (two patients) and PMR (two patients) based on imPERCIST due to the definition of the latter.

Finally, in 18 (60.0%) cases concordance was noted between the CT criteria (combined modified RECIST and RECIST 1.1) and three PET response classifications (EORTC, PERCIST, imPERCIST), while discordance was noted in 12 (40.0%), with moderate agreement (κ = 0.516 between CT criteria and EORTC, κ = 0.529 between CT criteria and PERCIST, κ = 0.544 between CT criteria and imPERCIST) noted between them for response classification (Table 4). Five (16.7%) of the present 30 patients were classified as CMR based on the EORTC, PERCIST, and imPERCIST criteria, which was not demonstrated by CT criteria (combined modified RECIST and RECIST 1.1).

### 3.3. Progression Free Survivals (PFS)

**Figure 3:**
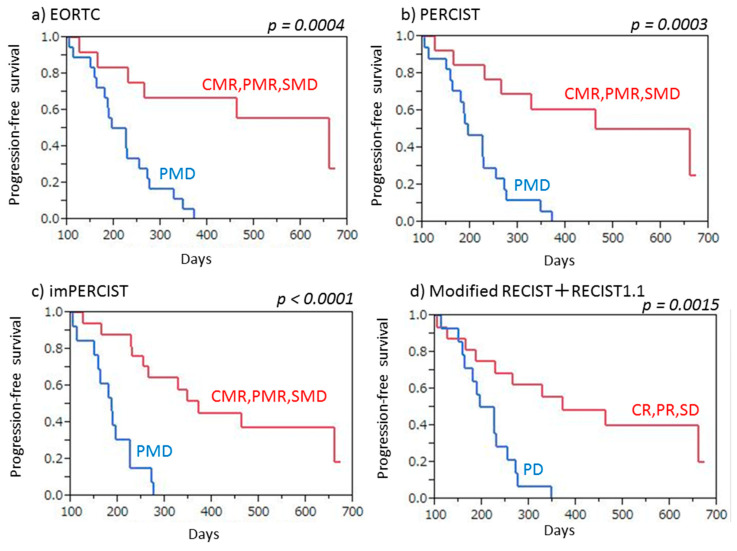
Progression-free survival (PFS) of malignant pleural mesothelioma patients treated by nivolumab therapy, with and without progression. (**a**) EORTC demonstrated that patients with no progression (CMR/PMR/SMD) showed significantly longer PFS than those with PMD (*p* = 0.0004). (**b**) PERCIST demonstrated that patients with no progression (CMR/PMR/SMD) showed significantly longer PFS than those with PMD (*p* = 0.0003). (**c**) imPERCIST demonstrated that patients with no progression (CMR/PMR/SMD) showed significantly longer PFS than those with PMD (*p* < 0.0001). (**d**) Combined modified RECIST and RECIST 1.1 demonstrated that patients with no progression (CR/PR/SD) showed significantly longer PFS than those with PD (*p* = 0.0015).

**Figure 4:**
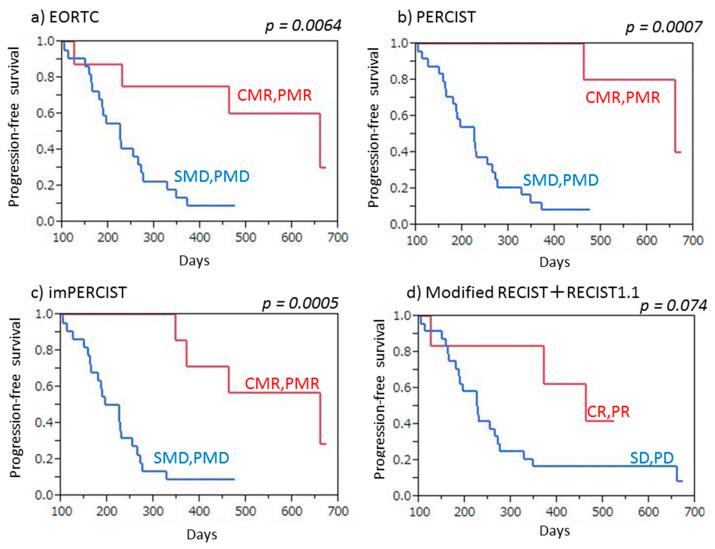
Progression-free survival (PFS) of malignant pleural mesothelioma patients treated by nivolumab therapy, with and without response. (**a**) EORTC demonstrated that responders (CMR/PMR) showed significantly longer PFS than non-responders (SMD/PMD) (*p* = 0.0064). (**b**) PERCIST demonstrated that responders (CMR/PMR) showed significantly longer PFS than non-responders (SMD/PMD) (*p* = 0.0007). (**c**) imPERCIST demonstrated that responders (CMR/PMR) showed significantly longer PFS than non-responders (SMD/PMD) (*p* = 0.0005). (**d**) Combined modified RECIST and RECIST 1.1 demonstrated that responders (CR/PR) tended to show longer PFS than non-responders (SD/PD), without a significant difference (*p* = 0.074).

Twenty-six (86.7%) of the 30 patients had progressive disease noted after a median period of 8.0 months (3.3–22.4 months). Both PET (EORTC, PERCIST, imPERCIST) and CT (combined modified RECIST and RECIST 1.1) criteria indicated a significantly longer PFS in patients with no progression (CMR/PMR/SMD, CR/PR/SD) as compared to those with PMD or PD (EORTC: *p* = 0.0004, PERCIST: *p* = 0.0003, imPERCIST: *p* < 0.0001, combined modified RECIST and RECIST 1.1: *p* = 0.0015) (Figure 3). Similarly, responders (CMR/PMR) based on PET criteria (EORTC, PERCIST, imPERCIST) showed significantly longer PFS than non-responders (SMD/PMD) (EORTC: *p* = 0.0064, PERCIST: *p* = 0.0007, imPERCIST: *p* = 0.0005), whereas use of CT criteria (combined modified RECIST and RECIST 1.1) showed that responders (CR/PR) had a tendency for longer PFS as compared to non-responders (SD/PD), though the difference was not significant (*p* = 0.074) (Figure 4).

### 3.4. Overall Survival (OS)

**Figure 5:**
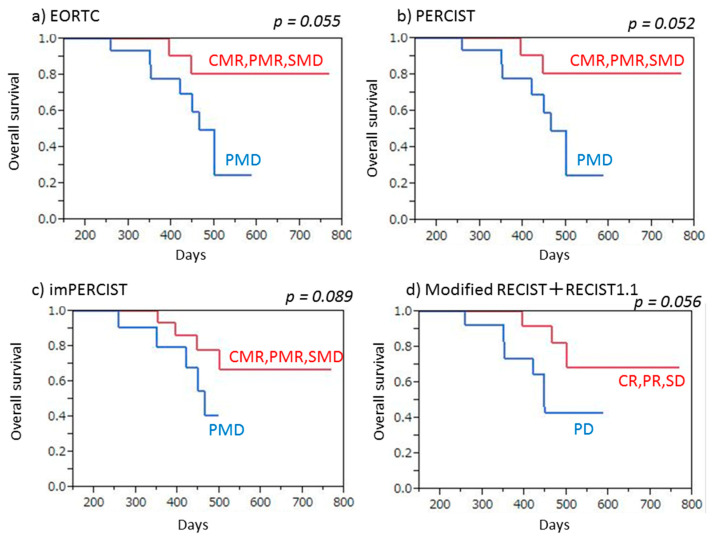
Overall survival (OS) of malignant pleural mesothelioma patients treated by nivolumab therapy, with and without progression. (**a**) EORTC demonstrated that patients with no progression (CMR/PMR/SMD) tended to show longer OS than those with PMD, without a significant difference (*p* = 0.055). (**b**) PERCIST demonstrated that patients with no progression (CMR/PMR/SMD) tended to show longer OS than those with PMD, without a significant difference (*p* = 0.052). (**c**) imPERCIST demonstrated that patients with no progression (CMR/PMR/SMD) tended to show longer OS than those with PMD, without a significant difference (*p* = 0.089). (**d**) Combined modified RECIST and RECIST 1.1 demonstrated that patients with no progression (CR/PR/SD) tended to show longer OS than those without PD, without a significant difference (*p* = 0.056).

**Figure 6:**
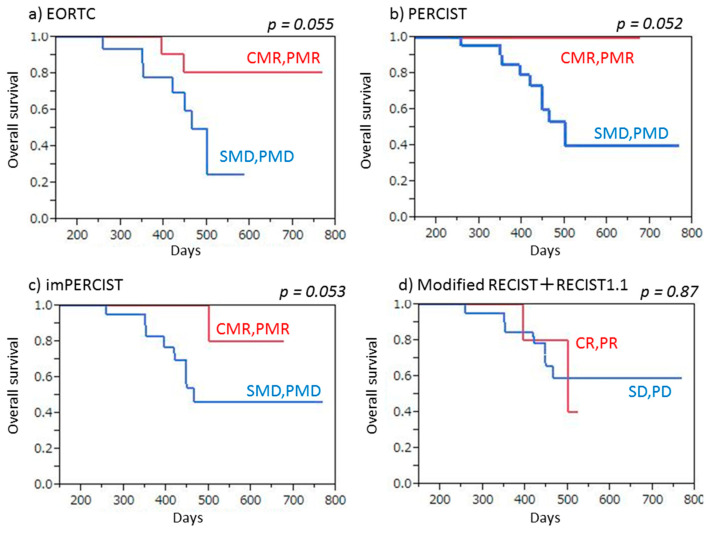
Overall survival (OS) of malignant pleural mesothelioma patients treated by nivolumab therapy, with and without response. (**a**) EORTC demonstrated that responders (CMR/PMR) tended to show longer OS than non-responders (SMD/PMD), without a significant difference (*p* = 0.055). (**b**) PERCIST demonstrated that responders (CMR/PMR) tended to show longer OS than non-responders (SMD/PMD), without a significant difference (*p* = 0.052). (**c**) imPERCIST demonstrated that responders (CMR/PMR) tended to show longer OS than non-responders (SMD/PMD), without a significant difference (*p* = 0.053). (**d**) Combined modified RECIST and RECIST 1.1 demonstrated no significant difference for OS between responders (CR/PR) and non-responders (SD/PD) (*p* = 0.87).

Nine (30.0%) of the 30 patients died from MPM after a median 14.9 months (5.8–25.6 months). Both PET (EORTC, PERCIST, imPERCIST) and CT (combined modified RECIST and RECIST 1.1) criteria indicated that patients without progression (CMR/PMR/SMD, CR/PR/SD) had a tendency for longer OS as compared to patients with PMD or PD (EORTC: *p* = 0.055, PERCIST: *p* = 0.052, imPERCIST: *p* = 0.089, combined modified RECIST and RECIST 1.1: *p* = 0.056), though the difference was not significant (Figure 5). Similarly, according to both PET (EORTC, PERCIST, imPERCIST) and CT (combined modified RECIST and RECIST 1.1) criteria, responders (CMR/PMR, CR/PR) showed longer OS than non-responders (SMD/PMD, SD/PD) (EORTC: *p* = 0.055, PERCIST: *p* = 0.052, imPERCIST: *p* = 0.053) without a significant difference, whereas CT criteria (combined mRECIST and RECIST 1.1) indicated that OS values for responders (CR/PR) and non-responders (SD/PD) were not different (*p* = 0.87) (Figure 6).

## 4. Discussion

This is the first known study to compare three FDG-PET/CT criteria (EORTC, PERCIST, imPERCIST) with CT criteria (combined modified RECIST and RECIST 1.1) used to evaluate tumor response to ICI therapy in patients with recurrent MPM as well as prediction of prognosis. All of the FDG-PET/CT and CT criteria analyzed were found to be accurate for both evaluation of tumor response and prediction of PFS in the present cohort, though the FDG-PET/CT criteria showed a slight superiority. FDG-PET/CT is known as an accurate tool for evaluating tumor viability, and the results are useful for clear diagnosis of CMR when a residual tumor does not have abnormal FDG uptake during or after treatment. We noted that the EORTC, PERCIST, and imPERCIST criteria classified five (16.7%) of the present 30 patients as CMR, which was not obtained with use of the contrast-enhanced CT criteria (combined modified RECIST and RECIST 1.1). Additionally, FDG-PET/CT findings are known to be accurate for detecting bone/muscle and tiny lymph node metastasis, as well as very small dissemination in a second FDG-PET/CT examination. This study found that the EORTC and PERCIST criteria were able to classify four and three more patients (10–13.3%) as PMD in comparison to contrast-enhanced CT results with use of the combined modified RECIST and RECIST 1.1 criteria. The number of PMD cases determined by imPERCIST was lower than that by the EORTC and PERCIST criteria, due to the imPERCIST definition (new lesions do not result in PMD and are included in the sum of SULpeak if they showed a higher uptake level than existing target lesions).

In summary, all three FDG-PET/CT criteria clearly judged more patients (16.7%) as CMR and two of those, EORTC and PERCIST, were able to judge more patients (10–13.3%) as PMD in comparison with CT criteria. For predicting PFS, the three FDG-PET criteria were superior to the CT criteria and imPERCIST demonstrated the highest rate of accurate prediction. It is considered that FDG-PET/CT might be a powerful tool for late (≥4 cycles) response assessment when evaluating ICI therapy and able to identify MPM patients who can most benefit from that. If MPM patients undergoing nivolumab were judged as non-PMD, nivolumab is continued. Unfortunately, MPM patients undergoing nivolumab were judged as PMD, alternative treatment (cisplatin/carboplatin and pemetrexed, pemetrexed, or irinotecan and gemcitabine) is tried in order to improve patient outcome.

Tumor infiltration by immune cells can delay tumor shrinkage or even cause a temporary increase in size (pseudoprogression), making assessment of tumor response to ICI treatment challenging. Although several criteria have been proposed for use with CT findings to determine response to that treatment, such as immune-related response criteria (irRC) [15], immune-related RECIST (irRECIST) [9], and immune RECIST (iRECIST) [16], as well as for use with FDG-PET results, including PET/CT criteria for early prediction of Response to Immune checkpoint inhibitor Therapy (PECRIT) [17], PET Response Evaluation Criteria for Immunotherapy (PERCIMT) [18], imPERCIST [10], and immune PERCIST (iPERCIST) [19], an optimal evaluation method has yet to be determined. Although pseudoprogression must be considered in the early phase following initiation of ICI treatment, that was not observed in any of the present 30 patients, probably due to late (≥4 cycles) response assessment.

There have been several articles demonstrating the usefulness of FDG-PET/CT for assessing the ICI therapeutic response, especially early response (2~4 cycles of ICI) in metastatic melanoma patients [10,17,18,20]. Cho et al. [17] analyzed PECRIT, which includes change in lesion size combined with change in FDG avidity shown by FDG-PET/CT after one cycle of ICI monotherapy (ipilimumab, nivolumab, or BMS-936559), in a study of 20 advanced melanoma patients. They found that criteria including SD shown by RECIST 1.1 and an SULpeak increase >15.5% in the hottest lesion shown by FDG-PET/CT were accurate for predicting treatment response after four months, with values for sensitivity, specificity, and accuracy of 100%, 93%, and 95%, respectively. In another study, PERCIMT, which uses absolute number of new lesions rather than changes in metabolic parameters (i.e., SUV) shown by FDG-PET/CT, was introduced by Anwar et al. [18] to evaluate 41 patients with metastatic melanoma after four cycles of ipilimumab. Those criteria, which include four or more new lesions <1 cm in functional diameter, were found to be accurate for clinical benefit prediction, with a sensitivity of 84% and specificity of 100%. Ito et al. [10] originally presented imPERCIST, in which the appearance of new lesions is not used to define PMD. Those authors noted that an increase in SULpeak sum of ≥30% in up to five measured lesions in FDG-PET/CT results accurately reflected PMD after 2–4 cycles of ipilimumab treatment in 60 metastatic melanoma patients. Although the significant and apparent superiority of FDG-PET/CT was not observed in our series, the potential reason may be biological difference between malignant melanoma and MPM, late (≥4 cycles) response assessment, or small sample size. With iPERCIST, Goldfarb et al. [19] introduced two new categories used for response to PMD, unconfirmed (UPMD) and confirmed (CPMD). Results of 28 non-small cell lung cancer patients who were receiving nivolumab were analyzed and indicated that any metabolic progression observed at eight weeks (after four cycles) should be confirmed by another FDG-PET/CT examination performed four weeks later, while they also noted that iPERCIST was useful for differentiation of responders from non-responders and OS prediction (*p* = 0.0003).

The present study has some limitations, including its retrospective nature, performance at a single center, and small sample size. Thus, generalization of the findings is limited and statistical errors are possible. To clarify the roles of FDG-PET/CT and CT for decision making, as well as predicting long-term outcomes in clinical settings a prospective multicenter trial with a larger cohort will be necessary. Additionally, the enrolled cohort was heterogeneous, as patients who underwent nivolumab monotherapy and received the second FDG-PET/CT examination after from four to 21 cycles were included; thus, confounding factors were likely introduced. The impact of PET/CT is primarily early within the course of treatment, because metabolic changes proceed volumetric changes [20]. This cannot be demonstrated in this study due to the very large and relatively late variation of the time points for the follow-up study. We are planning a prospective study to clarify both early and late response evaluation with less variation of the time to second and third FDG-PET/CT examinations from ICI treatment start, using three times of FDG-PET/CT examinations in MPM patients receiving ICI treatment Although we used four different PET/CT scanners, we harmonized PET quantitative values by a software, which can harmonize SUVs obtained with different PET/CT systems using phantom data [12]. Finally, irRC, irRECIST, iRECIST, and iPERCIST were not evaluated, because regular and follow-up CT and FDG-PET/CT examinations were not performed in every case.

## 5. Conclusions

In conclusion, results obtained with the use of three FDG-PET/CT (EORTC, PERCIST, and imPERCIST) and one CT (combined modified RECIST and RECIST 1.1) criteria were found useful to evaluate tumor response to ICI therapy as well as prediction of progression in recurrent MPM patients. In comparison with CT, all three FDG-PET/CT criteria judged a greater percentage of patients (16.7%) as CMR, while two (EORTC, PERCIST) judged a greater percentage (10–13.3%) as PMD. For predicting PFS, the three FDG-PET criteria were superior to the CT criteria, and imPERCIST demonstrated the highest rate of accurate prediction. Further validation in a prospective study with a larger cohort is warranted.

## Figures and Tables

**Table 1:** Study population characteristics.

Variable	Total Patients (*n* = 30)	%
Sex		
Male	24	80.0%
Female	6	20.0%
Age		
Mean	68.1 ± 7.2	
Range	46–77	
Histological subtypes		
Epithelial	24	80.0%
Sarcomatoid	4	13.3%
Biphasic	2	6.7%
Initial cStage		
I	9	30.0%
II	3	10.0%
III	14	46.7%
IV	4	13.3%
Previous treatment		
First line (Pemetrexed + cisplatin/carboplatin)	13	43.3%
First line + Second line (Pemetrexed)	3	10.0%
First line + Second line (Irinotecan + Gemcitabine)	2	6.7%
First line + Surgery	5	16.7%
First line + Surgery + second line (Pemetrexed + cisplatin/carboplatin)	5	16.7%
First line + Surgery + second line (Pemetrexed + cisplatin) + third line (Irinotecan + Gemcitabine)	1	3.3%
First line + Surgery + Second line (Pemetrexed)	1	3.3%

Data are presented as numbers.

**Table 2:** Comparison of treatment response assessments in EORTC criteria and PERCIST.

	EORTC Criteria				
	PMD	SMD	PMR	CMR	Total
PERCIST					
PMD	17	0	0	0	17
SMD	1	4	2	0	7
PMR	0	0	1	0	1
CMR	0	0	0	5	5
Total	18	4	3	5	30

Data are presented as numbers. Abbreviations: EORTC: European Organization for Research and Treatment of Cancer, PERCIST: Positron Emission Tomography Response Criteria in Solid Tumors, PMD: progressive metabolic disease, SMD: stable metabolic disease, PMR: partial metabolic response, CMR: complete metabolic response.

**Table 3:** Comparison of treatment response assessments in imPERCIST and two other PET citeria (EORTC criteria and PERCIST).

	EORTC Criteria					PERCIST				
	PMD	SMD	PMR	CMR	Total	PMD	SMD	PMR	CMR	Total
imPERCIST										
PMD	13	0	0	0	13	13	0	0	0	13
SMD	3	4	2	0	9	2	7	0	0	9
PMR	2	0	1	0	3	2	0	1	0	3
CMR	0	0	0	5	5	0	0	0	5	5
Total	18	4	3	5	30	17	7	1	5	30

Data are presented as numbers. Abbreviations: EORTC: European Organization for Research and Treatment of Cancer, PERCIST: Positron Emission Tomography Response Criteria in Solid Tumors, imPERCIST: immunotherapy-modified Positron Emission Tomography Response Criteria in Solid Tumors, PMD: progressive metabolic disease, SMD: stable metabolic disease, PMR: partial metabolic response, CMR: complete metabolic response.

**Table 4:** Comparison of treatment response assessments in CT criteria (combined modified RECIST and RECIST1.1) and three PET criteria (EORTC criteria, PERCIST, imPERCIST).

	EORTC Criteria					PERCIST					imPERCIST				
	PMD	SMD	PMR	CMR	Total	PMD	SMD	PMR	CMR	Total	PMD	SMD	PMR	CMR	Total
CT criteria															
PD	13	0	1	0	14	13	1	0	0	14	11	2	1	0	14
SD	4	4	1	1	10	3	5	1	1	10	2	6	1	1	10
PR	1	0	1	4	6	1	1	0	4	6	0	1	1	4	6
CR	0	0	0	0	0	0	0	0	0	0	0	0	0	0	0
Total	18	4	3	5	30	17	7	1	5	30	13	9	3	5	30

Data are presented as numbers. Abbreviations: EORTC: European Organization for Research and Treatment of Cancer, PERCIST: Positron Emission Tomography Response Criteria in Solid Tumors, imPERCIST: immunotherapy-modified Positron Emission Tomography Response Criteria in Solid Tumors, PMD: progressive metabolic disease, SMD: stable metabolic disease, PMR: partial metabolic response, CMR: complete metabolic response, PD: progressive disease, SD: stable disease, PR: partial response, CR: complete response.
